# 

**Published:** 1884-09-20

**Authors:** 


☛ Friends, send in the amounts due, and oblige the managing editor. The aggregate of these is important. R. C. WORD.

### EDITORIAL NOTICES.

SURGICAL INFIRMARY.—See the card of Prof. J. McF. Gaston in reference to a Surgical Infirmary in Atlanta.

SEE the advertisement of H. Franklin. Mr. Franklin has the character of a safe and enterprising business man—try him.

DISEASES OF WOMEN.—We invite attention to the advertisement of Drs. Taliaferro & Noble in reference to their Infirmary for the treatment of Uterine Diseases.

DR. T. F. RUMBOLD, former editor of St. Louis Medical and Surgical Journal, is now in Europe, getting data for a revised edition of his work entitled, *The Hygiene and Treatment of Catarrh.*

SOUTHERN MEDICAL COLLEGE.—The next session of this school will open on Tuesday, the 7th of October. The introductory lecture will be delivered by Prof. W. P. Nicolson, at eleven o'clock a. m. of that day, at the College building. The Profession and citizens generally are invited to be present.

CHOLERA.—The disease is spreading in Europe. About four thousand deaths are reported in France up to this date, and over three thousand in Italy. In Naples the onslaught of the disease was sudden and violent, and the percentage of mortality fearful.

### *RECEIPTED.*

Receipts will appear in our next.

